# Age-dependent diagnostic and treatment response prediction of TBII and TSI in Graves’ orbitopathy: integration with orbital MRI biomarkers

**DOI:** 10.3389/fimmu.2025.1657160

**Published:** 2025-12-15

**Authors:** Cheng Song, Xiao Wang, Qintao Ma, Genfeng Yu, Yan Zhu, Zimeng Huang, Tian Chen, Kai Huang, Yuanping Hai, Haixiong Chen, Yongbo Duan, Jie Shen

**Affiliations:** 1 Department of Endocrinology and Metabolism, The Eighth Affiliated Hospital, Southern Medical University (The First People’s Hospital of Shunde), Foshan, Guangdong, China; 2 The Second School of Clinical Medicine, Southern Medical University, Guangzhou, China; 3 Guangdong Engineering Technology Research Center of Metabolic Disorders Interdisciplinary Precision Prevention and Digital Healthcare, The Eighth Affiliated Hospital, Southern Medical University (The First People’s Hospital of Shunde), Foshan, Guangdong, China; 4 Department of Radiology, The Eighth Affiliated Hospital, Southern Medical University (The First People’s Hospital of Shunde), Foshan, Guangdong, China; 5 Department of Ophthalmology, The Eighth Affiliated Hospital, Southern Medical University (The First People’s Hospital of Shunde), Foshan, Guangdong, China

**Keywords:** antibodies, Graves’ orbitopathy (GO), MRI, comparison, activity, treatment response

## Abstract

**Background:**

Thyrotropin-binding inhibitory immunoglobulin (TBII) is involved in the pathogenesis of Graves’ orbitopathy (GO). Although thyroid-stimulating immunoglobulin (TSI) may offer superior diagnostic or prognostic value, its utility remains incompletely defined.

**Methods:**

This retrospective study included 177 consecutive patients with GO, comprising 128 newly diagnosed cases and 49 individuals with a history of intravenous methylprednisolone (IVMP) therapy. All participants underwent standardized evaluations, including endocrine assessments, ophthalmic examinations, and orbital magnetic resonance imaging (MRI). MRI was used to quantify the maximum signal intensity ratio of the extraocular muscles (SIR max) and the volume of the extraocular muscles (EMV). TBII levels were measured using a third-generation competitive-binding immunoassay, and TSI levels were assessed using a bridge-based chemiluminescence immunoassay. Treatment response to combined IVMP and mycophenolate mofetil (MMF) was evaluated in a subgroup of 70 newly diagnosed patients.

**Results:**

In newly diagnosed patients, the TSI positivity rate was significantly higher than that of TBII (*P* < 0.001). Notably, only within this subgroup did both antibodies show a positive correlation with the clinical activity score (CAS) (TBII: r=0.354, *P* < 0.001, TSI: r=0.323, *P* < 0.001) and SIR max (TBII: r=0.234, *P* = 0.008; TSI: r=0.175, *P* = 0.048). Multivariate analysis identified age (β=0.297, *P* = 0.002), TBII (β=0.365, *P* < 0.001), and TSI (β=0.325, *P* = 0.003) as independent factors associated with CAS. An age-stratified analysis demonstrated stronger correlations between antibody levels and CAS in patients older than 45 years (TBII-CAS: r=0.410; TSI-CAS: r=0.426), with correspondingly higher areas under the curve (AUC) for identifying active disease (TBII: 0.736; TSI: 0.760). In evaluating treatment response to IVMP combined with MMF, higher baseline TSI levels (OR = 1.086, 95% CI: 1.014–1.163), elevated SIR max (OR = 9.205, 95% CI: 1.072–79.053), and lower HDL levels (OR = 0.033, 95% CI: 0.003–0.346) were independently associated with poor outcomes. In contrast, TBII did not retain independent predictive value in this treatment context.

**Conclusion:**

In newly diagnosed patients with GO, both TBII and TSI levels were associated with disease activity, with their diagnostic value being more pronounced in older individuals. TSI demonstrated a higher positivity rate than TBII and served as an independent predictor of treatment response to IVMP combined with MMF.

## Introduction

Graves’ orbitopathy (GO), an autoimmune condition affecting approximately half of patients with Graves’ disease (GD), significantly impairs quality of life and psychosocial well-being. In 3%–5% of cases, the condition progresses to severe, sight-threatening optic neuropathy (1). Pathological studies have demonstrated high expression of thyroid-stimulating hormone receptors (TSHR) in orbital tissues and fibroblasts (2, 3). Anti−TSH receptor autoantibodies (TRAb), which serve as a critical biomarker for GO, bind to TSHR and activate downstream inflammatory pathways, including the PI3K-mTOR and PKA-cAMP signaling cascades. This molecular interaction promotes the accumulation of hyaluronic acid, adipogenesis, and lacrimal gland enlargement, ultimately to orbital tissue remodeling. The resulting changes give rise to various clinical manifestations of GO, including eyelid retraction, periorbital edema, proptosis, diplopia, lacrimation, and orbital pain (4).

Significant advances have been made in the detection of TRAb. The third-generation TRAb assay, which utilizes TSHR-coated ELISA plates, is the most widely employed method, achieving specificity and sensitivity exceeding 97% (5, 6). However, this assay measures total thyrotropin-binding inhibitory immunoglobulin (TBII) and does not distinguish between functionally stimulating antibodies (thyroid-stimulating immunoglobulin, TSI) and blocking antibodies (TBAb). Although TSI represents the truly pathogenic antibody subset, its measurement has traditionally been limited by procedures that are complex, time-consuming, and costly. Recently, a novel bridge-based TSI binding assay has been developed, which directly quantifies TSI levels by exploiting the formation of a “bridge” between two distinct TSHR molecules. This assay is highly specific for the active receptor conformation, although it may also detect a subset of blocking antibodies in addition to TSI (7). It has demonstrated superior concordance with clinical diagnoses of GD compared with conventional TBII assays and shows a positive correlation with the TSI bioassay (8–10).

Quantification of TSI specifically assesses the stimulatory component of TBII and may provide enhanced diagnostic sensitivity for GD and related autoimmune thyroid disorders. However, whether TSI outperforms TBII in evaluating GO activity, severity, or treatment response remains a matter of debate (11–14). Clinical manifestations of GO also exhibit marked age-related heterogeneity, with older patients frequently presenting with more severe disease. While specific biomarkers, such as CD3+ CD4+ T cells, have shown potential in age-stratified analyses (15, 16), it remains unclear whether TBII or TSI levels exhibit similar age-dependent variations in their associations with GO features.

In addition, both TBII and TSI hold promise as biomarkers of treatment response. Previous studies, including recent comparisons of TBII, bridge-based TSI, and bioassay-based TSI, have demonstrated their utility in predicting outcomes following IVMP monotherapy (17–20). Furthermore, the lipid profile, particularly low-density lipoprotein (LDL) levels, has been implicated in immune dysregulation and may serve as a predictor for the response to IVMP in GO patients (21). However, despite the recommendation of IVMP and mycophenolate mofetil (MMF) combination therapy as first-line treatment for moderate-to-severe active GO in the 2021 EUGOGO guidelines (22), whether these biomarkers can predict treatment outcomes remains unexplored.

The clinical activity score (CAS) is a standard tool for assessing GO activity; however, studies suggest that it may not fully capture disease activity, particularly in Asian populations, where low CAS scores can coincide with progressive clinical manifestations (23, 24). Quantitative orbital MRI provides a valuable objective complement to clinical assessment. During active inflammation, increased signal intensity within the extraocular muscles on fat-suppressed sequences (such as T2-Dixon)—correlates with the severity of inflammation. This can be quantified using signal intensity ratios relative to reference tissues such as cerebral white matter (25, 26) with the maximum value recorded as the maximum signal intensity ratio (SIR max). Additionally, increased extraocular muscle volume (EMV) has been associated with greater disease severity in GO (27, 28). Lacrimal gland involvement is also common, and the maximum cross-sectional area of the lacrimal gland (LG area) is recognized as a highly sensitive imaging marker of disease activity (29).

Therefore, this retrospective study has two objectives: (1) to comprehensively evaluate the correlations of TBII and TSI levels with clinical manifestations of GO and quantitative orbital MRI parameters (SIR max, EMV, LG area), and to investigate whether these correlations are age dependent; and (2) to compare the prognostic value of baseline TBII and TSI levels in predicting treatment outcomes following IVMP-MMF combination therapy.

## Materials and methods

### Patients enrolled

This retrospective study included consecutive patients evaluated for GO at the Endocrinology and Metabolism Department of our institution between January 2021 and March 2025. Data were extracted from the center’s electronic medical record system. GO diagnosis was confirmed by the Bartley criteria (30). The exclusion criteria were as follows: (1) absence of laboratory tests including thyroid function, TSI, TBII, or blood lipid levels; (2) unavailable or unclear MRI data; (3) systemic immunosuppressive therapy within the preceding 3 months (15); (4) history of orbital decompression surgery or radioactive iodine treatment; (5) concurrent significant ocular disease (e.g., glaucoma, severe cataracts) or other systemic autoimmune diseases; and (6) age <18 years.

**Figure 1:**
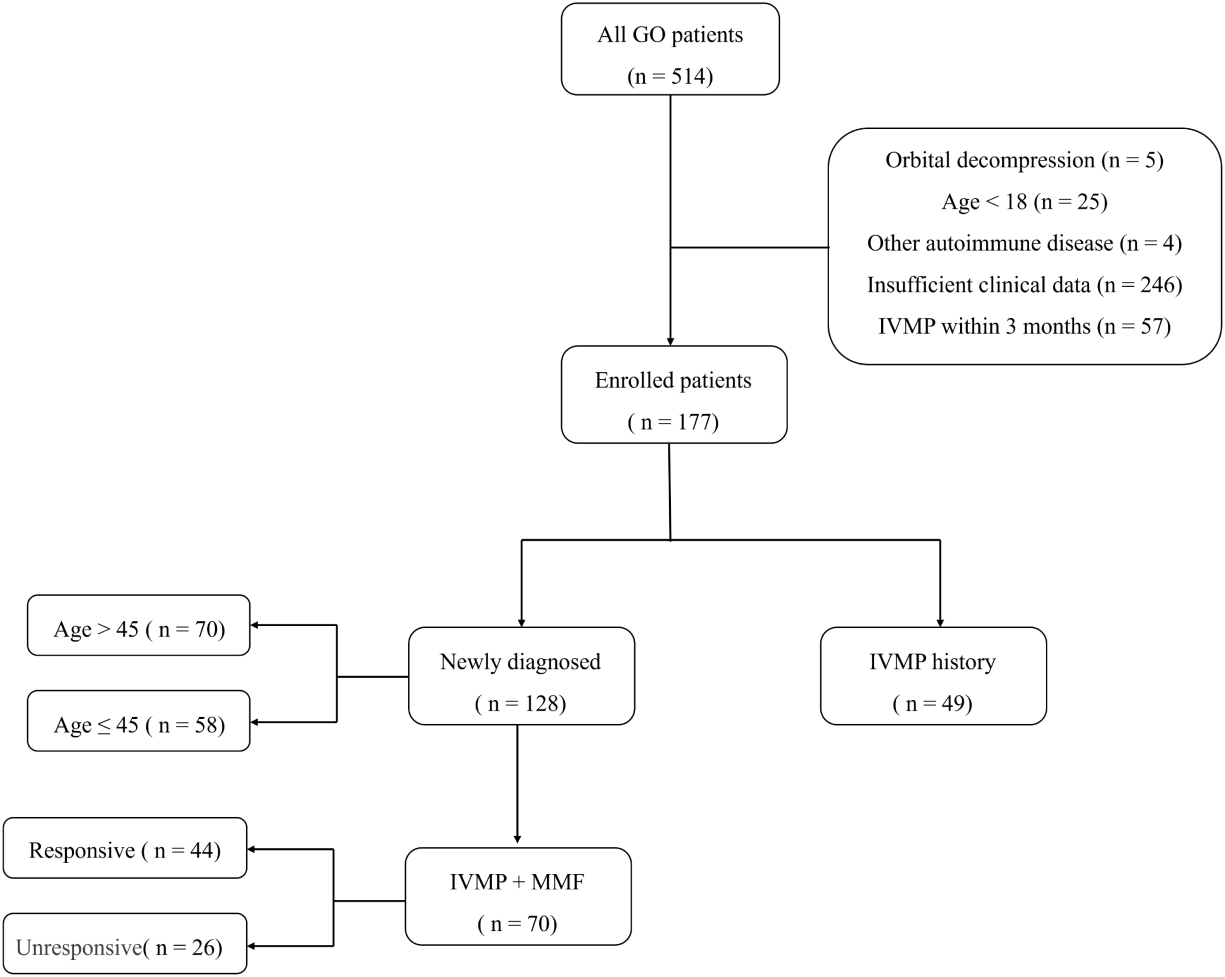
Study flow diagram.

The final study cohort comprised 177 eligible patients. A subgroup of 128 patients was newly diagnosed with orbitopathy, defined as having received no prior GO-specific therapy except for local therapies (e.g., eye lubricants). The status of their thyroid disease varied: among them, 90 patients were currently receiving antithyroid drugs (ATDs), 31 patients were newly diagnosed with GD, 6 patients had discontinued ATDs within the preceding six months, and 1 patient presented with hypothyroidism. The remaining 49 patients had received intravenous methylprednisolone (IVMP) therapy more than 3 months before enrollment. For treatment response analysis, the combination therapy protocol consisted of IVMP and oral MMF, which was administered to 70 treatment-naïve patients. IVMP was administered in a tapering regimen: a starting dose of 0.5 g per week for the initial phase, followed by a reduced dose of 0.25 g per week, with a total cumulative dose of 4.5 g. MMF was co-administered at a daily oral dose of 1.0 g for 6 months (**Figure 1**).

This study was conducted in accordance with the ethical standards of the Declaration of Helsinki and was approved by the Ethics Committee of the Eighth Affiliated Hospital of Southern Medical University (20201207). Written informed consent was obtained from each participant.

### Magnetic resonance imaging parameters assessment

**Figure 2:**
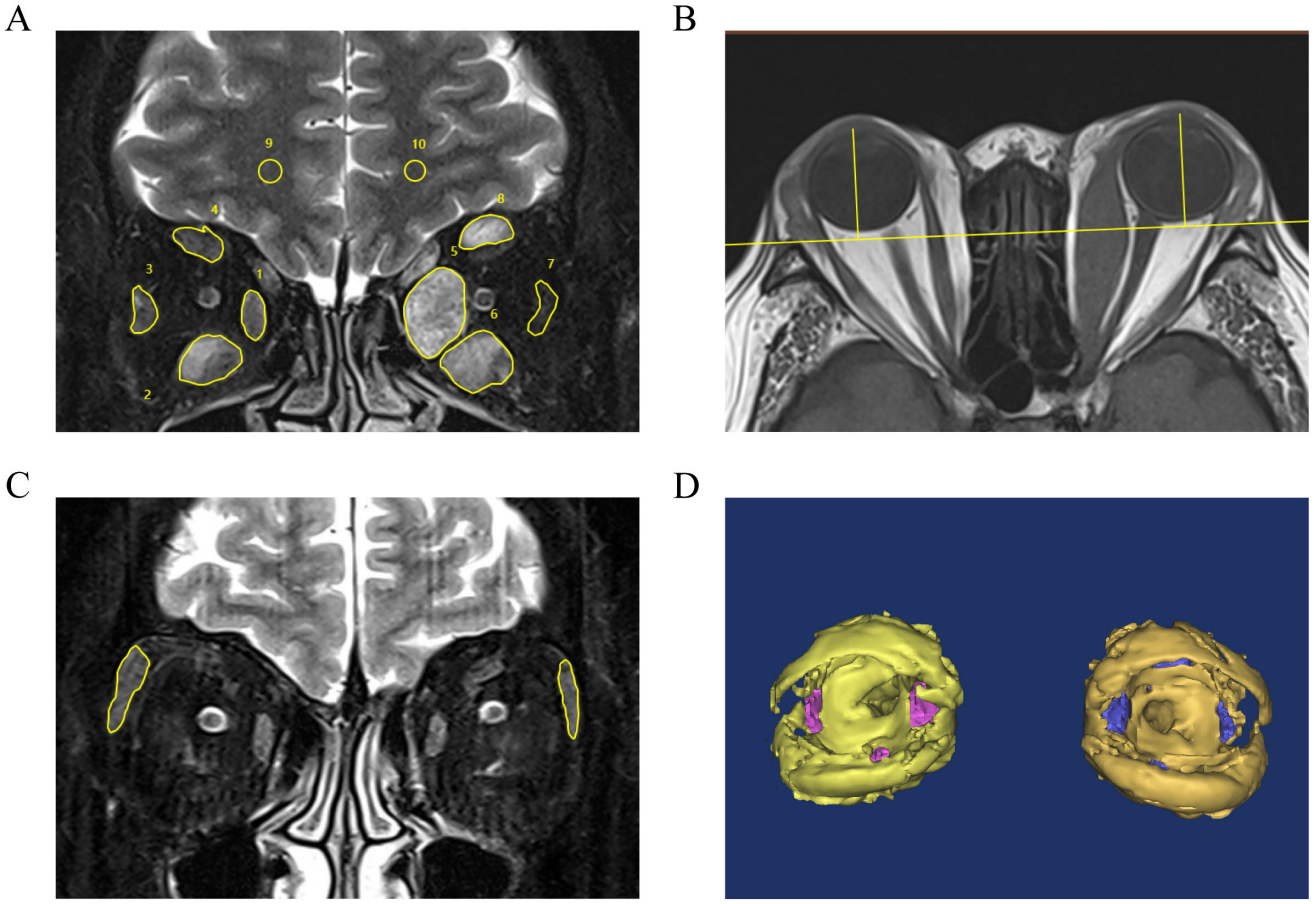
Orbital MRI acquisition and quantitative measurements. **(A)** Manual segmentation of EOMs and cerebral white matter (reference region) by radiologists. The most severe inflammation was identified in images taken 3, 6, and 9 mm posterior to the globe, and EOMs were outlined to measure signal intensity. SIR max was calculated as the highest value among (SI _EOMs_/SI _white matter_) for individual muscles. The SIRs of the left internal rectus, superior rectus, external rectus, and inferior rectus were 2.56, 2.42, 0.53, and 1.96, respectively; thus, the SIR max=2.56. **(B)** On the section that bisects the lens, the perpendicular distance from the corneal apex to the line connecting the bilateral zygomatic processes was defined as proptosis (left eye: 24.34 mm; right eye: 24.93 mm). **(C)** In the coronal series, the image in which the LG appeared the largest was chosen, and the area of the LG was manually enclosed (left: 33.4 mm^2^; right eye: 56.5 mm^2^). **(D)** Reconstruction of orbital fat and EOM and the volume of the soft tissues were calculated by MIMICs automatically (left eye: EOM volume=9.98 cm^3^, orbital fat volume=22.54 cm^3^). GO, Graves’ orbitopathy; EOMs, extraocular muscles; SIR, signal intensity ratio; LG, lacrimal gland.

All patients underwent a 3.0T orbital MRI examination (Siemens Healthcare, Germany). Sequences included axial T1-weighted turbo spin-echo (T1-TSE), coronal T2-Dixon, and 3D sagittal T1 magnetization-prepared rapid gradient echo. Detailed acquisition parameters and reconstruction methods have been previously published (31). Regions of interest on coronal T2-Dixon sequences were independently delineated by a junior radiologist with 6 years of experience and a senior radiologist with over 20 years of experience, both of whom were blinded to the patients’ clinical information. On images 3, 6, and 9 mm posterior to the globe, cross-sectional contours of the extraocular muscles (EOMs) showing the most severe inflammation were outlined to measure signal intensity. SIR max was calculated relative to the ipsilateral cerebral white matter. Additionally, contours of the lacrimal glands were outlined to determine the LG area. Proptosis was defined as the perpendicular distance from the corneal apex to the line connecting the bilateral zygomatic processes on T1-TSE images (**Figures 2A–C**) (32, 33). Inter-rater reliability was assessed using the intraclass correlation coefficient (ICC), with values above 0.75 indicating good agreement. The final results were calculated as the average of both assessments. Orbital fat and EOM volumes were quantified using Mimics software (Mimics 15.0; Materialize NV, Leuven, Belgium) for 3D reconstruction performed by a physician with 6 years of orbital reconstruction experience (**Figure 2D**). The reconstructions were reviewed and revised by the senior radiologist and the senior ophthalmologist to ensure accuracy and consistency.

### Clinical data collection

Disease activity was assessed using the seven-point scoring system recommended by the 2021 EUGOGO guidelines, with a clinical activity score (CAS) ≥ 3 indicating the active phase. Assessments were conducted independently by an endocrinologist with 9 years of experience and an ophthalmologist with 10 years of experience. Any discrepancies in their evaluations were resolved by a senior endocrinologist with more than 20 years of experience.

To further explore the role of the antibodies in GO, we analyzed whether baseline TBII/TSI levels could serve as indicators for IVMP combined with MMF therapy. A beneficial response was defined as meeting either of the following clinical criteria (18, 20): 1) achievement of a total CAS <3 in both eyes, or 2) an improvement of ≥ 2 points in one eye without concomitant deterioration in the fellow eye. In cases where clinical assessment based on CAS alone yielded equivocal results, a second MRI was performed for comparative evaluation. The final treatment response was adjudicated by a multidisciplinary team comprising specialists from endocrinology, ophthalmology, and radiology. Motility disorders were evaluated using Gorman scores: no diplopia (absent), diplopia only when the patient was tired or awakening (intermittent), diplopia at extremes of gaze (inconstant), and continuous diplopia in the primary and reading position (constant) (34).

Blood samples were obtained from all participants within 72 h before the baseline MRI scan. Serum samples from all patients were assayed for thyroid function and lipid profiles, including TBII (YHLO Biotech, Shenzhen, China), free triiodothyronine (FT3), serum free thyroxine (FT4), thyrotropin (TSH), thyroperoxidase antibodies (TPOAb), thyroglobulin (Tg), thyroglobulin antibody (TgAb) (Beckman-Coulter, Brea, CA, USA), triglyceride, total cholesterol (CHOL), high-density lipoprotein (HDL), and low-density lipoprotein (LDL) (Zybio Inc, Chongqing, China). TSI levels were measured using a chemiluminescence method (IMMULITE 2000 TSI; Siemens Healthcare Diagnostics, Llanberis, UK), with results provided by a laboratory accredited by the College of American Pathologists. The normal range were as follows: FT3: 3.53–7.37 pmol/L, FT4: 7.98–16.02 pmol/L, TSH: 0.56–5.91 mIU/L, TgAb: 0–4.9 IU/mL, TPOAb: 0–9 IU/mL, Tg: 1.15–130.77 μg/L, CHOL: 2.33–5.69 mmol/L, triglyceride: 0.56–1.70 mmol/L, HDL: 0.90–1.45 mmol/L, LDL: 0.10–3.10 mmol/L. TBII: 0–1.71 IU/L, TSI: 0–0.55 IU/L. The TBII and TSI kits had an upper detectable limit of 40 IU/L, and the results were recorded as 40 IU/L when values exceeded 40 IU/L.

### Statistical analysis

All variables were tested for normality using the Kolmogorov–Smirnov test and were presented as median (interquartile range, IQR) or mean ± standard deviation, depending on their distribution. Spearman’s rank correlation coefficient was used to assess correlations between variables. The Fisher’s exact test was used to compare the proportions of positive clinical characteristics. Simple linear regression or logistic regression was used to screen independent variables, and those with *P* < 0.1 were included in multiple regression analysis. The DeLong test was used to compare receiver operating characteristic (ROC) curves. Sensitivity rates of the two assays were compared in the GO group using the McNemar test. Because HDL was higher in the responsive group, the values were multiplied by −1 to place the curve above the reference line. Student’s t-test or the Mann–Whitney U test was applied, based on the normality of the independent variable, to identify differences between the two groups. P-values in the multiple linear regression analysis were adjusted using the Bonferroni correction. Statistical analyses were performed using SPSS 26 (IBM) and MedCalc 20.022 (MedCalc Software Inc.). β and adjusted P-value were calculated using R software (R Core Team, version 4.3.2). Statistical significance was defined as *P* < 0.05.

## Results

### Relationship between TBII/TSI and GO activity

Among the newly diagnosed group (n=128), the mean age was 45.50 ± 13.30 years, and 51.56% of the participants were men. The median disease duration was 5 months, and the median CAS was 3 points. In the IVMP history group (n=49), 42.86% were men, with a median disease duration of 9 months and a median CAS of 4. The ICCs for SIR max, proptosis, and LG area measurements were excellent (0.955, 0.971, and 0.946, respectively). Patient characteristics are presented in **Table 1**.

**Table 1:** Baseline demographical and clinical features of the patients.

Characteristic	Newly diagnosed group	IVMP history group
Number of patients	128	49
Age (y)	45.50 ± 13.30	45.02 ± 10.97
Sex (male, %)	66 (51.56%)	21 (42.86%)
Smokers	Non: 89 Ex: 6 Current: 33	Non: 37 Ex: 7 Current: 5
Alcohol consumption	9 (7.03%)	2 (4.08%)
Duration (m)	5.00 (2.00, 9.75)	9.00 (6.00, 13.00)
BMI	23.00 (21.23, 25.13)	24.10 (21.20, 26.59)
CAS	3 (2, 4)	4 (2, 5)
Gorman	1 (0, 2)	1 (0, 2)
FT3 (pmol/L)	5.68 (4.79, 7.17)	5.42 (4.78, 6.20)
FT4 (pmol/L)	11.99 (9.42, 18.25)	11.42 (9.30, 14.87)
TSH (mIU/L)	0.036 (0.003, 1.723)	0.926 (0.013, 2.751)
TBII (IU/L)	7.30 (2.24, 23.45)	6.92 (2.72, 15.63)
TSI (IU/L)	4.54 (1.81, 13.35)	4.97 (0.92, 10.55)
TPOAb (IU/mL)	34.80 (1.40, 227.10)	7.70 (0.60, 46.55)
TgAb (IU/mL)	0.90 (0.10, 46.68)	0.20 (0.10, 0.95)
Tg (μg/L)	25.39 (1.92, 123.98)	36.49 (10.49, 104.26)
CHOL (mmol/L)	4.86 (3.98, 5.66)	5.24 ± 1.05
Triglyceride (mmol/L)	1.17 (0.92, 1.68)	1.32 (1.02, 1.66)
HDL (mmol/L)	1.28 (1.09, 1.48)	1.33 (1.16, 1.54)
LDL (mmol/L)	2.73 (2.13, 3.36)	2.88 (2.46, 3.44)
Proptosis (mm)	19.38 ± 2.81	21.60 ± 3.59
SIR max	1.63 (1.23, 1.93)	1.67 (1.14, 1.97)
EMV (cm^3^)	3.67 (2.88, 4.73)	4.50 (3.66, 6.04)
OFV (cm^3^)	16.39 (14.27, 18.43)	19.50 (15.73, 22.47)
LG area (mm^3^)	68.38 57.84, 80.10)	74.65 (65.85, 88.48)

BMI, body mass index; CAS, clinical activity scores; FT3, free triiodothyronine; FT4, serum free thyroxine; TSH, thyrotropin; TBII, thyrotropin-binding inhibitory immunoglobulin; TSI, thyroid stimulating immunoglobulins; TPOAb, thyroperoxidase antibodies; TgAb, thyroglobulin antibody; Tg, thyroglobulin; CHOL, total cholesterol; HDL, high-density lipoprotein; LDL, low-density lipoprotein; SIR max, maximum of signal intensity ratio; EMV, extraocular muscle volume; OFV, orbital fat volume; LG, lacrimal gland; IVMP, intravenous methylprednisolone.

Within the newly diagnosed cohort, TBII levels showed a strong correlation with TSI levels (r=0.798, *P* < 0.001). Both TBII and TSI levels correlated positively with SIR max (TBII; r=0.234, *P* = 0.008, TSI: r=0.175, *P* = 0.048), and CAS (TBII: r=0.354, *P****<***0.001, TSI: r=0.323, *P****<***0.001). TBII levels were also positively correlated with EMV (r=0.182, *P* = 0.004). TSI levels showed positive correlations with proptosis (r=0.175, *P*= 0.049) and LG area (r=0.187, *P*= 0.035). Neither TBII nor TSI levels were correlated with the Gorman diplopia score (*P*= 0.941 and *P* = 0.780, respectively). Univariate regression analysis identified age, gender, smoking status, FT3, TPOAb, LDL, TBII, and TSI as candidates for multiple linear regression. Higher age (β=0.253, *P* = 0.015) and LDL levels (β=0.295, *P* = 0.004) were independently associated with increased EMV. Age (β=0.410, *P* < 0.001) was independently associated with elevated SIR max. For CAS, increased age (β=0.297, *P* = 0.002), TBII (β=0.365, *P* < 0.001), and TSI (β=0.325, *P* = 0.003) emerged as significant independent predictors (**Table 2**). Multiple regression analysis revealed no significant associations between TBII or TSI and LG area or proptosis (data not shown).

**Table 2:** Multivariate regression analyses of TRAb/TSI and activity parameters in GO.

Characteristic	EMV			SIR max			CAS		
	β	*P* value	*P*. adj	β	*P* value	*P*. adj	β	*P* value	*P*. adj
Age	0.253	0.002	**0.015**	0.410	<0.001	**<0.001**	0.297	<0.001	**0.002**
Gender	−0.265	0.009	0.085	−0.081	0.433	1.000	−0.310	0.002	**0.022**
Smoking 1	−0.009	0.918	1.000	0.065	0.441	1.000	−0.193	0.020	0.178
Smoking 2	−0.003	0.977	1.000	0.085	0.407	1.000	−0.008	0.933	1.000
FT3	−0.101	0.230	1.000	−0.055	0.529	1.000	−0.004	0.961	1.000
TBII	0.218	0.010	0.087	0.202	0.020	0.179	0.365	<0.001	**<0.001**
TPOAb	0.107	0.196	1.000	−0.002	0.982	1.000	0.003	0.966	1.000
LDL	0.295	<0.001	**0.004**	0.158	0.066	0.595	0.166	0.045	0.404
Age	0.279	<0.001	**0.005**	0.435	<0.001	**<0.001**	0.341	<0.001	**<0.001**
Gender	−0.245	0.017	0.154	−0.063	0.549	1.000	−0.277	0.007	0.065
Smoking 1	−0.007	0.931	1.000	0.067	0.438	1.000	−0.191	0.024	0.215
Smoking 2	0.012	0.906	1.000	0.099	3.453	1.000	0.022	0.828	1.000
FT3	−0.079	0.350	1.000	−0.034	0.697	1.000	0.027	0.751	1.000
TSI	0.172	0.051	0.462	0.159	0.085	0.725	0.325	<0.001	**0.003**
TPOAb	0.090	0.307	1.000	−0.017	0.856	1.000	−0.039	0.657	1.000
LDL	0.292	<0.001	**0.006**	0.155	0.740	0.666	0.164	0.052	0.471

FT3, free triiodothyronine; TBII, thyrotropin-binding inhibitory immunoglobulin; TSI, thyroid-stimulating immunoglobulins; TPOAb, thyroperoxidase antibodies; LDL, low-density lipoprotein; SIR max, the maximum signal intensity ratio of extraocular muscles; EMV, extraocular muscle volume; CAS, clinical activity score; P. adj, adjusted P value.

Smoking was set as a dummy variable; Smoking1 and Smoking2 represent ex-smokers and current smokers, respectively, compared with non-smokers.

Gender was assigned as a binary categorical variable (1=male; 2=female).

P. adj was calculated by the Bonferroni method.

Bold values indicate statistical significance.

A significant serological discordance was observed between the TBII and TSI assays in the newly diagnosed group. The TSI positivity rate was significantly higher than that of TBII (92.97% *vs*. 78.91%, *P* < 0.001). Analysis based on disease activity further confirmed the superior sensitivity of TSI. Among patients with active GO (CAS ≥ 3), the TSI positivity rate was significantly higher than that of TBII (97.53% *vs*. 86.42%, *P* = 0.012). This trend was also evident in patients with inactive disease (CAS <3), where TSI positivity remained significantly higher (85.11% *vs*. 65.96%, *P* = 0.022) (**Table 3**).

**Table 3:** Distribution of GO cases with positive or negative tests in two tests.

Patients	TSI (+)/TBII (+)	TSI (+)/TBII (−)	TSI (−)/TBII (+)	TSI (−)/TBII (−)	*P* value
Newly diagnosed group					
Total	98 (76.56%)	21 (16.41%)	3 (2.34%)	6 (4.68%)	**<0.001**
CAS ≥ 3	69 (85.19%)	10 (12.35%)	1 (1.23%)	1 (1.23%)	**0.012**
CAS <3	29 (61.70%)	11 (23.40%)	2 (4.26%)	5 (10.64%)	**0.022**
IVMP history group					
Total	39 (79.59%)	5 (10.20%)	2 (4.08%)	3 (6.12%)	0.453
CAS ≥ 3	28 (80.00%)	2 (5.71%)	2 (5.71%)	3 (8.57%)	1.000
CAS <3	11 (78.57%)	3 (21.42%)	0	0	0.250

TSI, thyroid-stimulating immunoglobulins; TBII, thyrotropin-binding inhibitory immunoglobulin; IVMP, intravenous methylprednisolone; CAS, clinical activity score.

Bold values indicate statistical significance.

The clinical profile of the TBII-negative/TSI-positive subgroup (n=21) was characterized by mild to moderate disease, with a median CAS of 2, a median proptosis of 20.42 mm, and a mean age of 43.33 years. Notably, 57.1% (12/21) of these patients did not present with diplopia. In contrast, the IVMP history group exhibited a strong correlation between TBII and TSI levels (r=0.758, *P* < 0.001), with no significant difference in positivity rates between the two assays (89.79% *vs*. 83.67%, *P* = 0.453) (**Table 3**). In this cohort, neither assay demonstrated significant correlations with orbital imaging parameters (SIR max, EMV, LG area) or clinical measures (proptosis, CAS) (**Table 4**). Additionally, neither assay effectively discriminated patients with active GO (AUC for TBII: *P=*0.550; AUC for TSI: *P* = 0.658).

**Table 4:** Correlation coefficient and P value of TBII/TSI with inflammatory biomarkers in the IVMP history group.

Characteristic	TBII	TSI
SIR max	r=0.171, *P=*0.241	r=0.171, *P=*0.241
EMV	r=0.191, *P=*0.190	r=0.277, *P=*0.054
CAS	r=0.130, *P=*0.372	r=-0.019, *P=*0.898
LG area	r=-0.212, *P=*0.144	r=-0.113, *P=*0.440
proptosis	r=-0.008, *P=*0.958	r=-0.026, *P=*0.861

SIR max, the maximum signal intensity ratio of extraocular muscles; EMV, extraocular muscle volume; CAS, clinical activity score; LG, lacrimal gland; TBII, thyrotropin-binding inhibitory immunoglobulin; TSI, thyroid-stimulating immunoglobulins.

### Age modifies the association between TBII/TSI and disease activity

**Figure 3:**
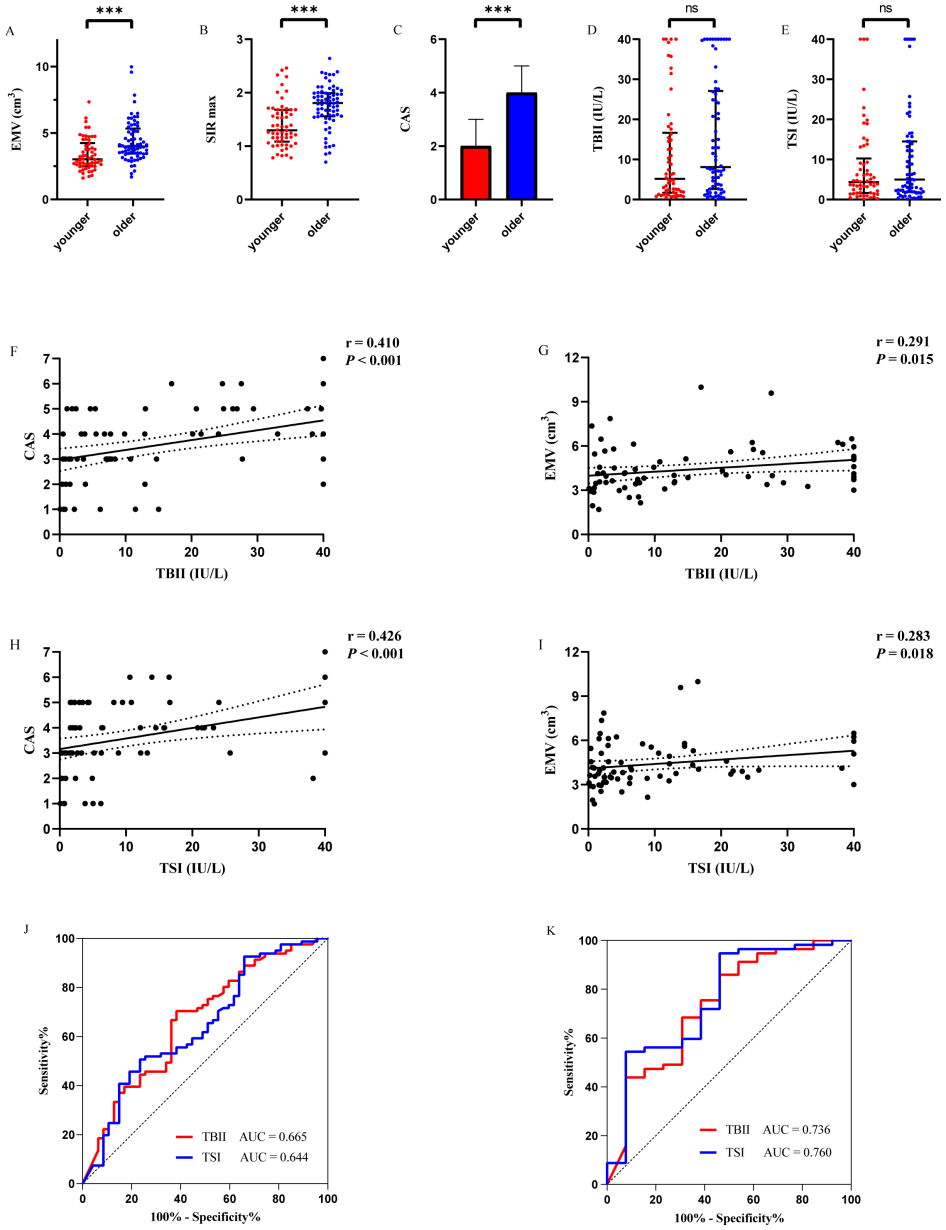
**(A-E)** Comparisons of EMV, SIR max, CAS, TBII, and TSI between younger (≤ 45 years) and older (> 45 years) subgroups in newly diagnosed patients (n=128). Although there was no significant difference in TBII/TSI levels between the two groups, EMV, SIR max, and CAS were higher in the elderly subgroup. **(F-G)** Correlation of serum TBII levels and CAS or EMV in patients > 45 years. The black lines represent fit plots. **(H-I)**, Correlation of serum TSI levels and CAS or EMV in patients > 45 years. The black lines represent fit plots. **(J)**. ROC curves for TBII and TSI discriminating active disease in all newly diagnosed patients (n=128). **(K)**. ROC curves for TBII and TSI discriminating active disease in patients > 45 years (n=70). The AUC values for each assay are indicated in the graphs. EMV, extraocular muscles volume; SIR max, maximum of signal intensity ratio of extraocular muscles; CAS, clinical activity scores; TBII, thyrotropin-binding inhibitory immunoglobulin; TSI, thyroid-stimulating immunoglobulins; ROC, receiver operating characteristic; AUC, area under the curve. ***P < 0.001. NS, no significance.

Given that age consistently emerged as an independent predictor of activity parameters (EMV, SIR max, and CAS) in multiple linear regression models within the newly diagnosed cohort, and in light of previous reports of age-related differences (35, 36), age-stratified analyses (≤ 45 years *vs*. > 45 years) were conducted to further examine the relationship between TBII/TSI levels and disease activity. Although baseline TBII (5.20 (1.71, 16.65) IU/L *vs*. 8.12 (2.66, 27.09) IU/L, *P* = 0.095) and TSI (4.36 (1.66, 10.25) IU/L *vs*. 4.98 (1.93, 14.50) IU/L, *P* = 0.585) levels did not differ significantly between age groups, patients aged ≤ 45 years exhibited significantly lower median CAS (2 (2, 3) *vs*. 4 (3, 5), *P* < 0.001), EMV (3.02 (2.51, 4.25) cm^3^*vs*. 3.99 (3.45, 5.33) cm^3^, *P* < 0.001), and SIR max (1.29 (1.08,1.68) *vs*. 2.00 (1.81, 2.28), *P* < 0.001) compared with those aged > 45 years (n=70) (**Figures 3A–E**). In the ≤ 45-year subgroup (n=58), neither TBII nor TSI demonstrated statistically significant associations with CAS (*P* = 0.428 and *P* = 0.471, respectively), EMV (*P* = 0.405 and *P* = 0.409, respectively), or SIR max (*P* = 0.983 and *P* = 0.866, respectively). In contrast, within the > 45-year subgroup, both TBII and TSI showed significant positive correlations with CAS (TBII: r=0.410, *P* < 0.001; TSI: r=0.426, *P* < 0.001) and EMV (TBII: r=0.291, *P* = 0.015; TSI: r=0.283, *P* = 0.018) (**Figures 3F–I**).

ROC curve analysis confirmed the age-dependent diagnostic utility of TBII and TSI. Specifically, neither antibody demonstrated a significant ability to discriminate active GO status in the ≤ 45-year group (both *P* = 0.076). In contrast, both TBII and TSI showed significant diagnostic performance in the > 45-year cohort (TBII: AUC = 0.736, *P* = 0.008; TSI: AUC = 0.760, *P* = 0.004) (**Figures 3J, K**). The AUC values for TBII and TSI did not differ significantly within the older subgroup (*P* = 0.540), indicating comparable diagnostic capacities.

### High baseline TSI predicts poor response to IVMP with MMF treatment

**Figure 4:**
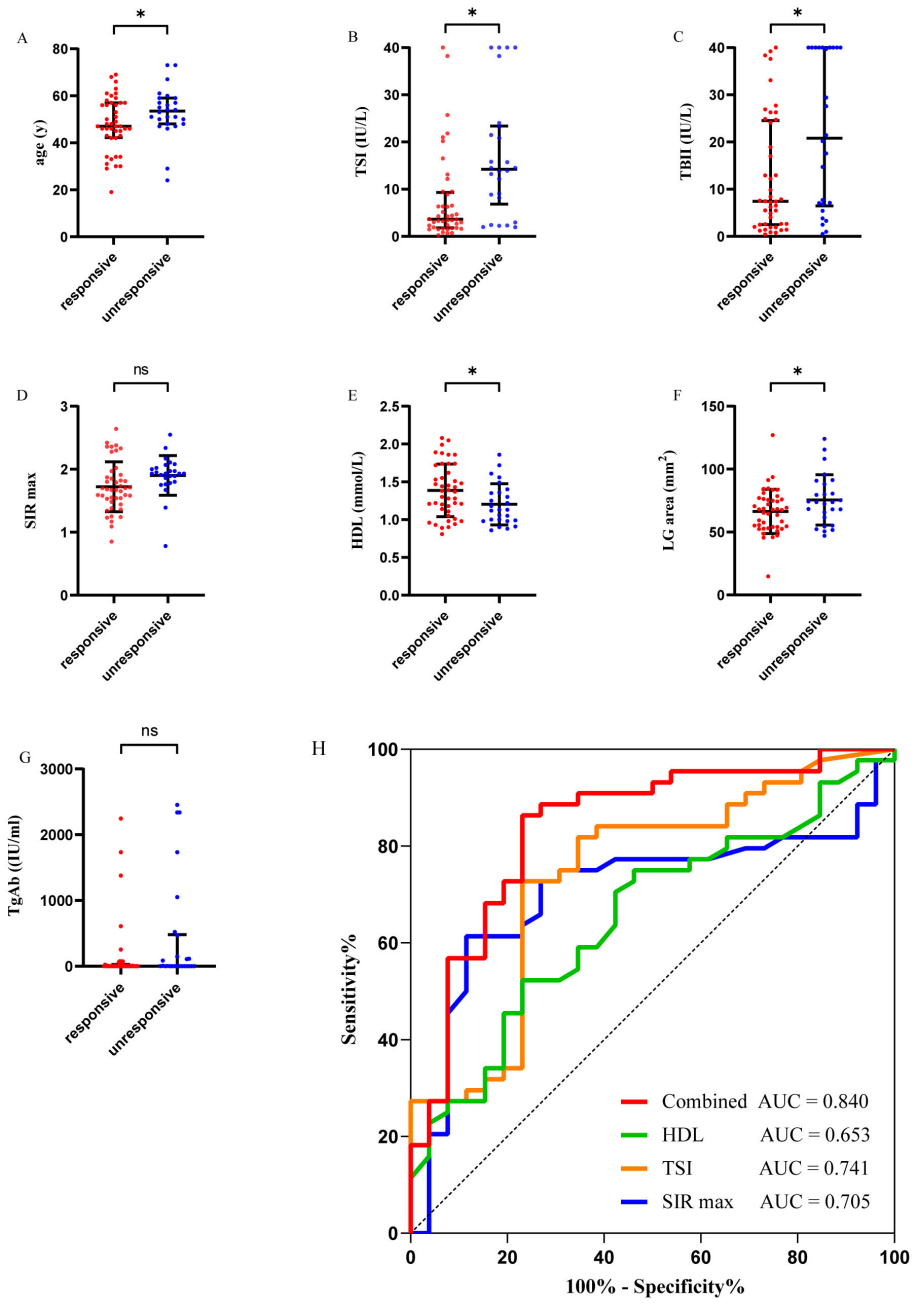
Predictors of treatment response identified by univariable logistic regression and their diagnostic performance. A-G. Comparison of clinical characteristics between the treatment-responsive and unresponsive groups. The presented variables—age **(A)**, TSI **(B)**, TBII **(C)**, SIR max **(D)**, HDL **(E)**, LG area **(F)**, and TgAb **(G)**—were selected based on a preliminary univariable logistic regression analysis. H, ROC curves evaluating the predictive performance of key parameters for discriminating treatment response. The AUC values for HDL, TSI, SIR max, and a combined parameter are indicated in the graph. TSI, thyroid-stimulating immunoglobulins; TBII, thyrotropin-binding inhibitory immunoglobulin; HDL, high-density lipoprotein; LG, lacrimal gland; SIR max, the maximum of signal intensity ratio of extraocular muscles. thyroglobulin antibody, TgAb; ROC, receiver operating characteristic; area under the curve, AUC. *P < 0.05. NS, no significance.

A total of 70 patients with moderate-to-severe active GO underwent IVMP for 12 weeks, followed by oral MMF for 24 weeks. Treatment outcomes were assessed upon completion of MMF therapy. Based on predefined response criteria, 44 patients were classified as responders and 26 as non-responders (**Table 5**). Variables identified as potentially significant in univariate screening—including LG area, SIR max, age, TgAb, HDL, TBII, and TSI—are visually summarized in **Figures 4A–G**, which presents scatter plots illustrating the distribution of each variable between the response groups.

**Table 5:** Clinical characteristics of IVMP with the MMF treatment group.

Characteristic	Responsive	Unresponsive	*P*
Number of patients	44	26	
Age (y)	47.00 (42.25, 57.00)	53.50 (48.00, 59.00)	**0.042**
Sex (male, %)	23 (52.27%)	17 (65.38%)	0.284
Smokers	Non: 30 Ex: 1 Current: 13	Non: 16 Ex: 1 Current: 9	0.825
Achol consumption	2 (4.55%)	2 (7.69%)	0.624
Duration (m)	3 (2, 6)	3 (2, 6)	0.699
BMI	23.19 ± 3.34	24.17 ± 3.76	0.261
CAS	3 (3, 4)	4 (3, 5)	**0.033**
FT3 (pmol/L)	5.29 (4.60, 6.75)	5.74 (4.98, 10.05)	0.217
FT4 (pmol/L)	11.66 (9.48, 15.73)	12.79 (9.33, 21.90)	0.395
TSH (mIU/L)	0.294 (0.009, 3.025)	0.027 (0.003, 2.606)	0.162
TBII (IU/L)	7.41 (2.50, 24.56)	20.82 (6.46, 40.00)	**0.012**
TSI (IU/L)	3.65 (1.81, 9.33)	14.20 (6.86, 23.39)	**0.001**
TPOAb (IU/mL)	55.60 (0.98, 175.45)	103.00 (4.30, 519.98)	0.133
TgAb (IU/mL)	1.00 (0.10, 26.85)	3.00 (0.10, 481.45)	0.292
Tg (μg/L)	24.47 (2.93, 69.31)	38.54 (1.72, 158.33)	0.508
CHOL (mmol/L)	5.05 ± 1.24	4.57 ± 1.43	0.141
Triglyceride (mmol/L)	1.27 (0.93, 1.77)	0.95 (0.85, 2.15)	0.422
HDL (mmol/L)	1.39 ± 0.35	1.20 ± 0.27	**0.025**
LDL (mmol/L)	2.74 (2.09, 3.34)	2.64 (1.81, 3.29)	0.395
Proptosis (mm)	20.36 ± 3.76	20.71 ± 2.88	0.679
SIR max	1.67 (1.49, 1.90)	1.93 (1.80, 2.03)	0.053
EMV (cm^3^)	3.89 (3.28, 4.78)	5.10 (3.85, 5.96)	**0.015**
OFV (cm^3^)	16.56 ± 3.00	17.03 ± 3.70	0.567
LG area (mm^3^)	66.36 ± 17.60	75.61 ± 20.01	**0.047**

BMI, body mass index; CAS, clinical activity scores; FT3, free triiodothyronine; FT4, serum free thyroxine; TSH, thyrotropin; TBII, thyrotropin-binding inhibitory immunoglobulin; TSI, thyroid-stimulating immunoglobulins; TPOAb, thyroperoxidase antibodies; TgAb, thyroglobulin antibody; Tg, thyroglobulin; CHOL, total cholesterol; HDL, high-density lipoprotein; LDL, low-density lipoprotein; SIR max, maximum of signal intensity ratio; EMV, extraocular muscle volume; OFV, orbital fat volume; LG, lacrimal gland.

Bold values indicate statistical significance.

These shortlisted variables were subsequently included in a multivariate logistic regression model for further analysis. Elevated TSI levels (odds ratio [OR]=1.086, 95% CI = 1.014-1.163), elevated SIR max (OR = 9.205, 95% CI = 1.072-79.053), and decreased HDL levels (OR = 0.033, 95% CI = 0.003-0.346) emerged as independent predictors of poor treatment response (**Table 6**). ROC analysis yielded the following AUC values for distinguishing non-responders: TSI: 0.741 (cutoff value=7.34 IU/L, *P* = 0.001), SIR max: 0.705 (cutoff value=1.74, *P=*0.004), and HDL: 0.653 (cutoff value=1.37 mmol/L, *P* = 0.033). Combining these three parameters significantly improved predictive performance, achieving a combined AUC of 0.840 (*P* < 0.001) (**Figure 4H**). The DeLong test confirmed that the combined model’s AUC was significantly higher than that of HDL (*P* = 0.006) and showed a trend toward being higher than SIR max (*P* = 0.050).

**Table 6:** Binary logistic regression of treatment response in GO.

Characteristic	OR	95% CI	*P*
LG area	1.032	0.995, 1.070	0.093
Age	1.046	0.980, 1.118	0.177
TgAb	1.000	0.999, 1.001	0.365
HDL	0.044	0.005, 0.422	**0.007**
SIR max	6.611	0.843, 51.830	0.072
TBII	1.034	0.989, 1.081	0.140
LG area	1.024	0.985, 1.065	0.226
Age	1.042	0.977, 1.112	0.212
TgAb	1.000	0.999, 1.001	0.838
HDL	0.033	0.003, 0.346	**0.004**
SIR max	9.205	1.072, 79.053	**0.043**
TSI	1.086	1.014, 1.163	**0.018**

LG, lacrimal gland; TgAb, thyroglobulin antibody; HDL, high-density lipoprotein; SIR max, maximum of signal intensity ratio; TBII, thyrotropin-binding inhibitory immunoglobulin; TSI, thyroid-stimulating immunoglobulins; OR, odds ratio; CI, confidence interval.

Bold values indicate statistical significance.

## Discussion

This study evaluated TBII levels using third-generation assays and quantified TSI using the IMMULITE 2000 system in newly diagnosed GO patients and those with a history of IVMP treatment. The results demonstrated that both TBII and TSI levels were positively correlated with orbital tissue involvement in the newly diagnosed group. Moreover, both assays exhibited comparable efficacy in differentiating active from inactive disease within this cohort, aligning with previous findings (12, 13, 20). This concordance is likely attributable to the predominance of TSI over TBAb in GO, as the latter is detectable in only a minority of cases (14, 37). Additionally, our analysis revealed that TSI showed significantly higher detection rates than TBII in both active and inactive subgroups within the newly diagnosed population. This enhanced sensitivity supports the superior utility of TSI for disease detection across the clinical spectrum of thyroid eye disease, particularly in inactive cases, which often present with subtle features such as mild extraocular muscle enlargement without overt clinical or inflammatory imaging findings. This observation further suggests that TSI may offer additional diagnostic value in differentiating GO from other orbital conditions with similar presentations, such as idiopathic orbital inflammation, allergic conjunctivitis, and IgG4-related disease, which may manifest with only mild eyelid swelling and LG enlargement on orbital imaging (38, 39).

In contrast, within the IVMP history group, neither TBII nor TSI showed significant correlations with disease activity parameters (SIR max, EMV, CAS), nor were they able to discriminate between active GO patients. Previous evidence indicates that IVMP significantly reduces TBII levels, with the most pronounced effects occurring within the first 3 months; however, this influence diminishes beyond 6 months and may even be followed by a rebound (40–42). In our cohort, the median interval since the last IVMP treatment was 4 (3, 7.5) months, placing patients in a transitional phase during which the uniformity of the initial treatment effect wanes, leading to considerable heterogeneity in individual antibody decay rates. It is this dissolution of a consistent treatment effect and the resulting interindividual variability—rather than a uniformly suppressed antibody state—that likely disrupts the stable correlation between antibody titers and the orbital disease burden. This phenomenon may account for the diminished predictive utility of both assays in this specific patient group.

A key finding of this study was the critical influence of age on the ability of TBII and TSI levels to reflect orbital involvement and disease activity. Previous studies have reported inconsistent correlations between TBII levels and CAS in younger cohorts (median/mean age <45 years) (13, 43–45), whereas significant associations have been consistently observed in older populations (median/mean age > 45 years) (14, 20, 46–48). Our data are consistent with this pattern, demonstrating improved biomarker accuracy in reflecting orbital involvement among older patients with GO. The mechanisms underlying this age-dependent effect warrant further consideration. Initial hypotheses proposed age-related differences in TSHR expression within orbital tissues; however, recent evidence has shown no significant correlation between orbital TSHR abundance and age (49). Instead, age-related functional differences in orbital fibroblasts (OFs)—the principal effector cells in GO—may contribute to this phenomenon. Notably, older patients with muscle-predominant GO exhibit distinct circular RNA expression profiles and differential pathway activation in OFs, suggesting that cellular responses to autoantibodies may be modulated by age-dependent molecular programs (50). Cytokine-stimulated OFs from older patients exhibit proliferative responses that correlate with clinical activity markers. In contrast, OFs from younger patients are predisposed to adipogenic differentiation, which is more closely associated with the development of proptosis (51). Furthermore, downstream signaling following TSHR activation involves cross talk with the insulin-like growth factor 1 receptor (IGF-1R), a pathway critically modulated by β-arrestin-1 and β-arrestin-2. While β-arrestin-2 primarily mediates TSHR desensitization, β-arrestin-1 enhances TSHR signaling activation (52). Notably, altered β-arrestin expression—particularly reduced β-arrestin-2 levels—has been implicated in aging-related conditions such as neurodegeneration and type 2 diabetes (53), suggesting that diminished β-arrestin-2 in older individuals may enhance the efficiency of TSHR-mediated inflammatory signaling.

Age-related changes in immune cell composition may also contribute to the observed effects. Age-associated B cells (ABCs), a unique subset that accumulates with aging, are characterized by a CD11c^+^T-bet^+^ phenotype and rely on TLR7/TLR9 signaling and Th1 cytokines, including IFN-γ and IL-21, for their differentiation (54, 55). These cells are enriched in autoimmune diseases, such as IgG4-related disease, where they promote inflammation through the production and secretion of autoantibodies and pro-inflammatory cytokines, including TNF-α and IFN-γ. In GO, ABCs may amplify TRAb-associated inflammation by promoting IgG4 synthesis—consistent with the elevated IgG4 levels observed in orbital tissues from patients with severe disease—and by secreting cytokines that activate OFs (56, 57). Additionally, ABCs can influence T-cell polarization toward a Th17 phenotype, thereby enhancing IL-17–mediated pro-inflammatory signaling and glycosaminoglycan production by fibroblasts (55, 58).

Regulatory T cells (Tregs), a key immunoregulatory CD4^+^ T-cell subset, also exhibit age-related functional decline, marked by reduced STAT3 activation and impaired suppression of IL-17–producing T cells. This diminished Treg-mediated control in older individuals permits the expansion of pathogenic T cells and exacerbates pro-inflammatory responses (59, 60). In GO, these dual alterations likely impair the regulation of TRAb-induced orbital inflammation, thereby amplifying tissue injury driven by TRAb signaling. Future studies should aim to elucidate the mechanisms underlying the cross talk between TRAbs and age-associated immune cell populations.

In addition, we evaluated the prognostic value of TBII and TSI for treatment outcomes following first-line therapy with IVMP combined with MMF in patients with moderate to severe active GO. Elevated TSI levels, increased SIR max on MRI, and reduced HDL concentrations emerged as significant independent predictors of poor treatment response, whereas TBII levels showed no significant prognostic utility. This finding diverges from prior studies assessing IVMP monotherapy, which reported predictive value for both TBII and TSI (20, 61). Several factors may account for this discrepancy. Most notably, our study evaluated a combination regimen of IVMP plus MMF, in contrast to IVMP monotherapy in earlier research. Additionally, our response assessment incorporated MRI parameters alongside CAS to adjudicate ambiguous cases, whereas some previous studies relied solely on CAS. Other methodological differences include a shorter median disease duration in our cohort (3 months), inclusion of a broader set of prognostic variables—such as imaging metrics and lipid profiles—in our multivariate model, and evaluation of sustained treatment response at 3 months post-IVMP (following MMF completion), rather than immediate post-treatment outcomes reported elsewhere.

Interestingly, recent studies have shown that HDL exerts immunomodulatory effects by suppressing the activation, proliferation, and cytokine production of CD4+ T lymphocytes (62), which constitute the most abundant lymphocyte population infiltrating orbital tissues in GO, exhibiting both chemotactic and pro-inflammatory characteristics (63). These findings highlight the importance of identifying biomarkers that reliably reflect disease severity, thereby facilitating more accurate outcome prediction.

Both TBII and TSI demonstrated only weak correlations with the total CAS, a result consistent with previous reports (46, 61). Several factors may account for these modest associations. First, GO disease activity is likely governed by a multifactorial process extending beyond TRAb titers, encompassing the local cytokine milieu, fibroblast responsiveness, and the well-established cross talk between the TSHR and IGF-1R signaling pathways (4, 64). Second, antibody concentrations measured in the peripheral circulation may not accurately reflect their bioactive levels within orbital tissues. Finally, in nearly half of all patients, hyperthyroidism precedes the onset of ocular symptoms (65), suggesting that peak antibody titers may occur before the onset of maximal clinical activity. Given the cross-sectional design of our study, these dynamic temporal dissociations may not have been adequately captured.

This study has several limitations. First, it employed a retrospective design, and TSI levels were not measured using bioassay methods, which may limit direct comparability with previous studies. Second, the disease duration was generally short among patients included in the analysis; as a result, its potential impact was not considered in the regression model. Finally, the sample size for the treatment response analysis was relatively small. Future multicenter, prospective studies with larger patient cohorts are warranted to improve the statistical power of longitudinal prognostic modeling.

In summary, in newly diagnosed GO patients, both TBII and TSI levels were associated with disease activity, with their diagnostic utility notably enhanced in older individuals. The bridge-based TSI assay demonstrated a higher positivity rate compared with TBII and emerged as an independent predictor of treatment outcome in patients receiving IVMP combined with MMF. However, no significant difference was observed between TSI and TBII in their ability to distinguish between active and inactive diseases.

## Data Availability

The raw data supporting the conclusions of this article will be made available by the authors, without undue reservation.
